# The (*FTO*) gene polymorphism is associated with metabolic syndrome risk in Egyptian females: a case- control study

**DOI:** 10.1186/s12881-017-0461-0

**Published:** 2017-09-16

**Authors:** Mina S. Khella, Nadia M. Hamdy, Ashraf I. Amin, Hala O. El-Mesallamy

**Affiliations:** 1 Biochemistry Department, Faculty of Pharmacy, Ain Shams University, African Union Organization Street, Abbassia, Cairo, 11566 Egypt; 2 Clinical Pathology and Laboratory Department, National Institute of Diabetes and Endocrinology (NIDE), Cairo, Egypt

**Keywords:** *FTO*, Obesity, SNP, T2DM, Alt, HDL-c, Metabolic syndrome, Serum lipids

## Abstract

**Background:**

Variations within fat mass and obesity associated (*FTO*) gene had crosstalk with obesity risk in European and some Asian populations. This study was designed to investigate *FTO* rs9939609 association with metabolic syndrome (MetS) as well as biochemical parameters as plasma glucose, serum triacylglycerol (TAG), total cholesterol (TC) and transaminases enzymes in Arab female population from Egypt.

**Methods:**

In order to achieve that, *FTO* gene rs9939609 (A < T) was genotyped using TaqMan SNP Genotyping Assay in a total of 197 females which were enrolled in this study. Fasting levels of serum insulin, lipid profile and plasma glucose, in addition to liver transaminases were measured. The association between the genotype distribution and MetS risk was evaluated using Chi-square and logistic regression tests in a case-control design under different genetic models.

**Results:**

The association of genotype distribution with MetS was significant (χ2 = 8.6/*P* = 0.014) with an increased odds ratio under dominant model (OR = 1.97, *P* = 0.029 and 95%C.*I* = 1.07–3.6) and recessive model (OR = 2.95, *P* = 0.017 and 95%C.*I* = 1.22–7.22). Moreover, (AA) subjects showed significant lower HDL-C levels (*P* = 0.009) when compared to (TT) ones. In addition, interestingly subjects with (AA) genotype have significantly higher ALT levels (*P* = 0.02) that remained significant after correction of major confounders as body mass index and serum triacylglycerols but not after conservative Bonferroni adjustment.

**Conclusions:**

The present study shows for first time that *FTO* gene rs9939609 is genetic risk factor for metabolic syndrome in Egyptian population which may help in understanding the biology of this complex syndrome and highlighted that this association may be through HDL-C component. The association of this genetic polymorphism with ALT levels needs to be studied in other populations with larger sample size.

## Background

Metabolic syndrome (MetS) is considered a major risk factor for cardiovascular diseases and stroke [1] in both developing and developed countries [2, 3] . The central obesity and type 2 diabetes mellitus (T2DM) are considered the major endophenotypes of MetS which are inheritable and genetically based [4–6]. Interestingly, Egypt had the highest mean female BMI in developing countries and even exceeded USA [7]. In Egypt, 22% of men while 48% of women were obese according to 2010 WHO estimate. The Middle East and North Africa Region are considered to have the highest prevalence of diabetes at 10.9***%*** [8]. The prevalence of MetS in Egyptian females has been reached about 45.8% [9]. The *FTO* (fat mass- and obesity-associated) gene is a good candidate gene for T2DM and MetS, since its possible link to obesity with genome wide association study based evidence [10]. In addition, human *FTO* gene (16q12.2) shows ubiquitous expression in adult and fetal tissues with the most significant high expression in hypothalamus [10] linking the FTO protein function with control of energy state [11]. FTO protein is one of the non-heme dioxygenase superfamily, which demethylate nucleic acids in a 2- oxoglutarate-dependent manner [11]. Despite several studies showed association of *FTO* gene polymorphism with obesity in different populations mainly of European descent [12–16], this came in contrast with other studies in which the *FTO* polymorphisms do not associate with BMI or obesity risk in consistent manner as in many populations with different ethnicity [16–19]. Moreover, The association of *FTO* with T2DM is still inconclusive [20]. The first intronic *FTO* variant rs9939609 was selected specifically in this study as it depicted the highest genotyping success rate among other obesity risk SNPs and has strong genome wide association evidence with obesity [10]. Furthermore, it is highly possible that this variant, even it is not the true functional variant, is in linkage disequilibrium with true functional ones either within *FTO* gene or nearby genes [16].

Therefore, this study was designed to investigate the association of *FTO* gene rs9939609 with metabolic syndrome and its endophenotypes in Arab female population from Egypt.

## Methods

### Study population

A total of random unrelated 197 subjects were studied. 92 with MetS were recruited from National Diabetes Institute – Cairo – Egypt versus 105 controls without MetS. The cases and controls were carefully chosen according the IDF definition of metabolic syndrome [21]. The true selection factors include obesity anthropometric measures (waist circumference ≥ 80 cm and BMI > 30 kg/m) plus any two of the following: blood glucose level (Fasting plasma glucose ≥100 mg/dl or previously diagnosed Type 2 diabetes), HDL-C (< 50 mg/dl or specific treatment for this lipid abnormality) and triacylglycerol (≥ 150 mg/dl or specific treatment for this lipid abnormality). All participants were subjected to full history and clinical data collection including blood pressure and anthropometric measures. There was a complete medical evaluation for each case with provided informed consent prior to inclusion in the study. Subjects diagnosed with severe acute infections, viral hepatitis‚ any endocrine disturbances or administrating sex hormones or corticosteroids for long period were excluded. All diabetic patients received only oral hypoglycemic drugs (metformin and sulfonyl urea agents) and no insulin and there was no statistical difference among patients regarding treatment agents. The initial population number was 207 females from which 10 females were excluded due to presence of liver anomaly. Since the MetS is a complex disease, the cases and controls were chosen carefully to minimize the effects of other non-genetic risk factors and were matched for dietary habits and exercise levels through asked questionnaire including dietary habits and exercising levels (the questionnaire included questions about the diet preference and whether it is carbohydrate rich, protein rich, vegetable rich or balanced one and results expressed as number and percentage and there was no significant difference between the cases and controls where the most of them used to eat carbohydrate rich diet with little protein and vegetables. Regarding the exercise levels, they were asked in the questionnaire in terms of exercising hours per day and there was also non-significant difference. In addition, any control with history of childhood obesity was excluded. Moreover, the age difference between the cases and controls was considered in the analysis of results and adjusted in all statistical tests. The Ethical Committee both of Faculty of Pharmacy, Ain Shams University, Cairo, Egypt and the National Diabetes Institute approved this study. Moreover, the study was performed in adherence to the Declaration of Helsinki Guidelines.

### Blood sampling

Whole blood samples (7–10 ml each) were drawn in the early morning after an overnight fasting period of at least 12 h. Blood Samples were collected in separate vacutainer tubes (BD Diagnostics, Franklin Lakes, NJ, USA): one containing disodium-ethlenediamine tetra-acetic acid (EDTA) for whole blood collection (3 ml) required for DNA extraction. A second plain vacutainer was used for serum collection that was divided into aliquots for measuring lipid profile, aminotransferases and fasting insulin. The third vacutainer was fluoride containing one for collecting plasma used for measuring fasting plasma glucose. Glucose and lipids profile parameters were assayed immediately; whereas, other aliquots were immediately frozen at (−80 °C) until subsequent assays were performed.

### Anthropometric and biochemical measurements

Anthropometric measurements included body mass index (BMI) (Kg/m^2^) and waist circumference (WC) cm. Non stretchable measuring tape was used to measure waist for the subjects while they are standing in erect relaxed position.

Biochemical measurements included plasma glucose was determined by using Dimension RxL analyzer (Dade Behring, Newark, DE) automated biochemistry analyzer. Serum total cholesterol (TC), triacylglycerol (TAG), high-density lipoprotein (HDL) cholesterol, aspartate aminotransferase (AST) and alanine aminotransferase (ALT) were determined by enzymatic methods using autoanalyser (Beckman synchron cx systems). Low-density lipoprotein (LDL) cholesterol was calculated by the Friedewald formula [22]. Serum insulin was measured using commercially available ELISA kit (Immunospec® kit, provided from Immunospec Corporation, Canoga Park, CA, USA). Homeostasis model assessment (HOMA-IR) calculated as: [(FBG X fasting insulin)/405] [23] and Quantitative insulin sensitivity check index (QUICKI) calculated as: 1/[log FI (μIU/ml) + log FBG (mg/dl)] [24] were used as a measure of insulin resistance.

### Genotyping

Whole Blood DNA was extracted using QIAamp DNA Mini Kit protocol (QIAGEN, Santa Clarita, CA). The extracted DNA was stored at −80 °C until used for genotyping assay. *FTO* gene genotyping rs9939609(A < T) was performed by TaqMan SNP Genotyping Assays (ABI; Applied Biosystems international, Foster City, CA) and using Real-Time PCR System (The Applied Biosystems® Step One Plus™**).** 10% selections of the samples were re-genotyped with 100% concordance to assure genotyping reproducibility.

### Statistical analysis

Statistical analyses were performed using windows based Statistical Package for Social Sciences (SPSS®) computer database version 17.0 and Microsoft Excel® version 2010.

Data were described as (Mean ± SD) for numeric variables, or median and range for numeric nonparametric variables, and number and percentage (%) for categorical variables.

The difference between the groups was analyzed using an independent Student’s *t*-test (for numeric parametric variables) and Mann-Whitney’s *U*-test (for numeric nonparametric variables) in case of only two groups. Comparisons between the 3 groups with respect to normally distributed numeric variables were done using the one-way analysis of variance (ANOVA). For significant tests, Bonforreni post hoc was performed for the pairwise comparisons. The Kruskal-Wallis test and the post hoc Bonferroni test were used to compare between groups regarding non-normally distributed numeric variables. Chi-square test was used to compare between the groups with respect to categorical data.

Conservative Bonferroni adjustment was applied for significant parameters (HDL-C and ALT) to avoid multiple testing error (adjusted *P* value at 0.01).

Genotype and allele frequency distributions were compared with the (χ2) test and Hardy–Weinberg equilibrium was computed using an online calculator [25].

Logistic regression was performed to evaluate associations of rs 9,939,609 SNP with risk of MetS by calculating the odds ratio and their adjusted values for age with their 95% confidence interval and corresponding *p*-value.

Multiple regression model was set to study the association of *FTO* polymorphism with ALT levels after correction of BMI and serum triglycerides.

*P*-values of <0.05 were considered significant. The power of study was calculated and reached nearly 70% taking significance level at 0.05, disease prevalence 48%, minor allele frequency 0.39 and average odds at 1.5 based on an online calculator (http://csg.sph.umich.edu) [26].

## Results

As shown in ‘Table 1’, there was significant difference in anthropometric and biochemical measurements between the study groups. The BMI, waist circumference, plasma glucose, HOMA-IR, TAG, TC and LDL-C were significantly higher, while HDL-C level was lower in the group with MetS vs control group.

**Table 1:** Characteristics of MetS cases (*n* = 92) and controls (*n* = 105)

Characteristic	MetS cases (*n* = 92)	Controls (*n* = 105)	*p*
Age, years, mean ± S.D	45.33 ± 9.56	44.74 ± 9.67	0.672^**c**^
BMI, Kg/m^2^, mean ± S.D	37.64 ± 5.7	26.07 ± 5.4	**<0.001** ^**a**^
Waist (cm),mean ± S.D	119.76 ± 10.96	89.68 ± 13.87	**<0.001** ^**a**^
TC (mg/dl), mean ± S.D	182.12 ± 44.52	165.42 ± 22.74	**0.001** ^**a**^
TAG (mg/dl), mean ± S.D	177.45 ± 84.06	110.06 ± 33.25	**<0.001** ^**a**^
HDL-C (mg/dl), mean ± S.D	38.51 ± 9.33	44.76 ± 8.07	**<0.001** ^**a**^
LDL-C (mg/dl), mean ± S.D	108.11 ± 39.44	98.58 ± 20.31	**0.038** ^**a**^
FPG (mg/dl), median(min-max)	161(64-361)	89(60-323)	**<0.001** ^**b**^
FSI(*μ*IU/mL), median(min-max)	13.39(2.9–30.3)	8.5(1-30)	**<0.001** ^**b**^
HOMA-IR, median(min-max)	4.48(0.77–20.46)	1.9(0.2-23.93)	**<0.001** ^**b**^
QUICKI, mean ± S.D	0.31 ± 0.04	0.35 ± 0.05	**<0.001** ^**a**^
FTO (rs9939609)			
TT, n(%)	24 (26.1)	43 (41)	
AT, n(%)	50 (54.4)	54 (51.4)	
AA, n(%)	18 (19.5)	8 (7.6)	**0.014** ^**c**^

Values are mean ± SD for parametric variables or median (range) for non-parametric variables. *BMI* Body mass index, *WC* Waist circumference, *T.C* Total cholesterol, *TAG* Triacylglycerol, *HDL-C* High density lipoprotein-cholesterol, *LDL-C* Low density lipoprotein-cholesterol, *FPG* Fasting plasma glucose, *FSI* Fasting serum insulin

^a^Independent T- test, two-sided *p* value >0.05 non-significant

^b^Mann–Whitney U test, two sided *p* value >0.05 non-significant

^c^Chi-square test, two-sided *p* value >0.05 non-significant

In the whole population the distribution of genotype for rs 9,939,609 variant were 34% TT, 52.8% TA and 13.2% AA. In the MetS group, the genotype distribution was 26.1% TT, 54.4% TA and 19.5% AA vs 41% TT, 51.4% TA and 7.6% AA in the control group, as shown in ‘Table 2’. This came in agreement with Hardy–Weinberg equilibrium (*P* > 0.05) in the whole population and in the individual study groups. The overall minor allelic frequency (MAF) of the risk allele A of *FTO* gene rs9939609 was (0.39).

**Table 2:** Association between FTO genotypes and MetS risk

	OR ^a^	95% CI	*p*
Dominant Genetic Model			
TT	Reference	Reference	
AT/AA	1.97	1.07, 3.6	**0.029**
Recessive Genetic Model			
AT/TT	Reference	Reference	
AA	2.95	1.22, 7.22	**0.017**

^a^ ORs, 95% CIs, and *p* values were calculated by Logistic regression

As depicted in ‘Table 2’, the dominant genetic model showed a 1.97-fold increase in the odds of MetS (95% C.*I* = 1.07–3.6, *p* = 0.029). Under recessive genetic mode, the AA genotype showed a 2.95-fold increase in the odds of MetS (95% C.*I* = 1.22–7.22, *p* = 0.017).

There was no significant difference in both anthropometric and biochemical measurements of the whole population among the different genotypes at all models (additive, dominant, and recessive) except for ALT and HDL-C levels. Regarding ALT, it was significantly higher in AA carriers (*P* = 0.02), as shown in ‘Table 3’. The association remained significant after correction of BMI and serum TAG. However, this association was abolished after applying the conservative Bonferroni adjustment for multiple testing. Regarding HDL-C levels, the AA genotype showed significantly lower levels (*P* = 0.009) and interestingly it remained significant after conservative Bonferroni adjustment.

**Table 3:** association between FTO (rs9939609) polymorphism and Clinical characteristics of subjects

Characteristics	FTO (rs9939609) Genotypes			*P*
	TT(*n* = 67)	AT(*n* = 104)	AA(*n* = 26)	
Age, years, mean ± S.D	46.3 ± 8.6	44.02 ± 9.8	45.65 ± 10.7	0.29 ^a^
BMI, Kg/m^2^, mean ± S.D	31 ± 8	31.37 ± 8.2	33.1 ± 7.2	0.51^a^
Waist (cm),mean ± S.D	103.2 ± 20.2	103.3 ± 20.1	106.8 ± 16.1	0.69^a^
TC (mg/dl), mean ± S.D	178.3 ± 33.1	172.4 ± 36.2	163.4 ± 38	0.18 ^a^
TAG (mg/dl), mean ± S.D	148.3 ± 73.8	138.6 ± 71.9	136 ± 58	0.62 ^a^
HDL-C (mg/dl), mean ± S.D	43.2 ± 10.7	42.2 ± 8	37 ± 8.5	**0.01** ^a^
LDL-C (mg/dl), mean ± S.D	105.4 ± 29.7	102.5 ± 32.7	99.3 ± 27.7	0.67 ^a^
FPG (mg/dl), median(min-max)	108(69–323)	100.5(60–361)	138.5(76–350)	0.2 ^b^
FSI(*μ*IU/mL), median(min-max)	10.5(2.4–30)	9.73(1–30.3)	12.2(2.9–29.3)	0.59 ^b^
HOMA-IR,median(min-max)	3.1 (0.6–23.9)	2.4 (0.2–20.45)	3.6 (1.29–20.46)	0.28 ^b^
QUICKI, mean ± S.D	0.33 ± 0.042	0.34 ± 0.06	0.32 ± 0.034	0.11 ^a^
AST(IU/mL), median(min-max)	15(4–53)	12.5(4–60)	17(8–58)	0.19 ^b^
ALT(IU/mL), median(min-max)	14(4–60)	11(4–57)	15.5(6–58)	**0.02** ^b^

*BMI* Body mass index, *WC* Waist circumference, *T.C* Total cholesterol, *TAG* Triacylglycerol, *HDL-C* High density lipoprotein-cholesterol, *LDL-C* Low density lipoprotein-cholesterol, *FPG* Fasting plasma glucose, *FSI* Fasting serum insulin

^a^one way ANOVA test, two-sided *p* value >0.05 non-significant

^b^ Kruskal-Wallis test, two-sided *p* value >0.05 non-significant

## Discussion

This is the first study to investigate the allele frequency and genotype distribution of *FTO* gene rs9939609 and its association with MetS including its endophenotypes of serum lipids in Egyptian female population. The overall MAF (A) in our study was (0.39) which is very similar to some European population as that of adult population from Spain which was (0.39) [12] and slightly lower than that of Italian population which was (0.48) [13]. Regarding Asian ancestry, a remarkably different MAF appeared which was (0.2) in Japanese population [27], (0.19) in Pakistani females [28] and (0.12) in Chinese population [29]. Our allele frequency results were also higher than that of black African population which was (0.35) [30] and slightly lower than the Brazilian one which was (0.435) [31] .

Despite MAF similarity in the current study with some European population, the genotypes distribution in our study groups was unique. In addition, our study revealed a positive association between *FTO* variant and MetS for the first time in Egyptian Arab female population. Al-Attar et al. (2008) reported in non-Caucasian population an association between rs9939609 *FTO* SNP and MetS risk that was dependent on obesity and HDL-C components. Moreover, the association was more prominent in men [32]. Our study declared association with MetS in cohort of Arab Egyptian Caucasian females that may be dependent partially on the association with lowered HDL-C levels. In agreement with our study, another positive association was observed in Han Chinese between the *FTO* SNP rs8047395 and MetS but with no significant associations with any endophenotypes of MetS [33]. Another important strength point of our study is that the cases and controls were carefully matched in other non-genetic risk factors of the MetS like the dietary habits and exercise levels which sheds more light on the impact of genetic factors. Regarding the association of *FTO* variation with lipid profile levels, our study showed non-significant association except for HDL-C levels. This came in contrast with other studies which showed non-significant association with all lipid profile [29, 34] or showed association with elevated triglycerides levels [35, 36]. However, it came in agreement with other studies depicted reduced HDL-c levels [36–38]. Moreover, the associations in other studies were more prominent in men [32, 39]. Subjects with AA genotype have higher BMI and WC compared to TA and TT carriers, however this difference didn’t reach statistical significance, may be due to relatively small sample size and different effect size in case of BMI. This is in agreement with three previous studies that didn’t detect association of rs9939609 *FTO* polymorphism with BMI and/or WC in a similar sample size [19, 35, 40]. In addition, there was association of *FTO* polymorphism with MetS in our study under both the dominant and recessive models unlike other studies like the one carried on Italian male and female population in which only the TA genotype showed association [41] and the one carried among Tunisian population [42]. Xu et al. (2014) showed significant association of *FTO* variations with some metabolic parameters under recessive model and even in higher sample size of our study [43]. We hypothesized that the association under recessive model was apparent here in our study may be due to association of (AA) subjects with lowered HDL-C levels compared to (TT) subjects. Those differences may be due to different ethnic group, different sample size, different study design or different population age.

The *FTO* association with MetS may be related to association with obesity [10], T2DM [44] and/or lipid abnormalities [32, 35]. The exact mechanism by which FTO protein is linked to obesity and T2DM is still completely unknown. Being highly expressed in the hypothalamus, FTO plays a key role in energy homeostasis [11]. Genetic variants of *FTO* were associated with increased energy intake [40, 45, 46]. Based on bioinformatics and in vitro studies *FTO* encodes for demethylase enzyme for DNA and RNA nucleotides especially RNA single strands [11, 47]. Changes in the DNA methylation state are significantly associated with many pathological diseases and abnormalities including obesity [48, 49]. For example, it is interesting that Indian children obesity was affected by vitamin B12 and folate maternal levels [50] indicating that methylation state plays a key role in risk contribution for obesity and related disorders [51]. This highlights that FTO link with obesity could be mediated by epigenetic changes in DNA methylation [52]. Moreover, the FTO plays a key role in the regulation of food intake through its function of sensing the levels of amino acids in the hypothalamus [53] which is known to affect orexigenic and anorexigenic pathways [54]. Very recently, it was shown that genetic variants within *FTO* gene are linked functionally to another distant gene called IRX3 which may link the association of *FTO* SNPs with both obesity and T2DM [55, 56]. Moreover, our study suggests for the first time an association of *FTO* polymorphism with high ALT levels that seems to be BMI independent, though the association was no longer statistically significant when corrected for multiple comparisons. Wu et al., 2010 hypothesized that the intronic genetic variants like rs9939609 affected the expression of FTO protein which may modulate adipogenesis in adipose tissue [57]. Interestingly nonalcoholic fatty liver disease (NAFLD) rat model in which the *FTO* overexpressed in liver showed increased oxidative stress and lipogenesis and was associated also with increased ALT levels [58]. This suggests an association of *FTO* genetic variation with ALT might be mediated through NAFLD, though participants with overt liver disease were excluded from our study. Another possibility is that FTO contributes directly to the regulation of hepatic metabolism [59]. Very recently, Guan et al. (2014) showed a significant association between ALT level and variants of some obesity-susceptible genes, but not *FTO*, either dependent or independent of BMI [60]. As a result this association with ALT needs to be validated in other populations with large sample size. We acknowledge the relative small sample size, however we are taking an advantage of the high frequency of the variant understudy in the Egyptian population, as reported to be 39% with unique distribution in the control and cases group which improves the power of study. Hopefully, our reported findings act as base for further future studies with larger sample size.

## Conclusions

this study highlighted for the first time a possible link between the *FTO* homozygous variant (AA) and the risk for MetS in Arab Egyptian female population that may be mediated through HDL-C lowered levels. Moreover, our study suggests a novel BMI and TAG-independent association of *FTO* genetic variation with elevated ALT levels that needs to be validated in other large sample sizes. We believe that this study hopefully helps in clarifying the pathophysiology and etiology of metabolic syndrome. Moreover, it will aid in early predicting those with high risk of developing metabolic syndrome which will result in better personalized strategies of treatment, nutrition or prevention. In addition our study, by linking *FTO* polymorphism with metabolic syndrome highlights the possible link between the *FTO* polymorphism with cardiovascular disorders.

## Abbreviations

ALT: Alanine aminotransferase
AST: Aspartate aminotransferase
BMI: Body mass index
FPG: Fasting plasma glucose
FSI: Fasting serum insulin
FTO: Fat mass and obesity associated
HDL-C: High density lipoprotein- cholesterol
LDL-C: Low density lipoprotein-cholesterol
MetS: Metabolic syndrome
SNP: Single nucleotide polymorphism
T.C: Total cholesterol
T2DM: Type 2 diabetes mellitus
TAG: Triacylglycerol
WC: Waist circumference
